# Patient Satisfaction with Postaural Incision Site

**DOI:** 10.1155/2014/851980

**Published:** 2014-09-24

**Authors:** George Barrett, Susanne Koecher, Natalie Ronan, David Whinney

**Affiliations:** Department ENT Head and Neck Surgery, Royal Cornwall Hospital, Truro TR1 3LJ, UK

## Abstract

*Introduction*. Controversy exists over the optimum incision placement when performing ear surgery via the postauricular approach. Little is known about the impact of incision placement on future comfort in wearing audio or visual aids or the effect on the minor auricular muscles cut in the approach. *Objective*. (1) To establish patient satisfaction with their postauricular surgical incision, and to establish the impact on comfort wearing hearing or visual aids. (2) To establish whether patients' voluntary ear movements were affected by surgery. *Materials and Methods*. In January 2014, questionnaires were sent to 81 patients who underwent mastoid surgery requiring a postauricular incision between January 2004 and December 2012. The incision placement was broadly the same for all patients as they were operated on by the same surgeon (or under his supervision). The incision is sited far posteriorly at the hairline. *Results*. 42 (52%) of the patients contacted responded. 80% of patients wearing glasses reported no discomfort or problems associated with their incision. 82% of patients who wear hearing aids were comfortable. Only 1 of the 5 patients who could move their ears preoperatively noticed a change afterwards. *Conclusion*. A hairline incision is well tolerated by most of the patients.

## 1. Introduction

Postaural incisions are well established in middle ear surgery and are used as an approach to combined approach tympanoplasty and cortical mastoidectomy. Due to the cosmetic advantage from their concealed position and direct access to the mastoid air cells, they also play an increasing role in cochlear implant surgery [1, 2]. They are also used in other fields of head and neck surgery, including resection of benign parotid gland tumours [3] and robotic-assisted neck dissection [4].

**Figure 1:**
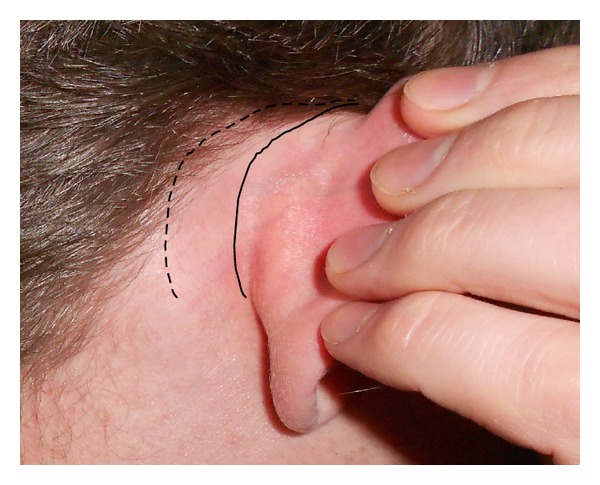
Photograph showing the locations of sulcal (solid line) and hairline (dashed line) postauricular incision for access to the mastoid.

There are two main surgical approaches for postaural incisions in the context of middle ear surgery. A curved incision can be made either in the postauricular sulcus (sulcus incision or in the groove incision) or along the hairline posterior to the sulcus (hairline incision or behind the groove incision), as shown in Figure 1.

There may be specific local factors such as skin lesions or previous scarring which dictate the precise site of surgery, but largely the incision location is based on surgeon preference and can vary considerably. After cutting through skin and subcutaneous tissue, the auricular muscles auricularis superior and posterior are generally divided to access and incise the periosteum overlying the mastoid bone. Depending on the surgery being performed a small amount of temporalis fascia may be harvested, but the underlying muscle is left intact.

Little is known about the role of these minor auricular muscles in humans. Infant mammals are believed to instinctively retract their auricles to comfortably position the head when nursing [5]. The postauricular reflex also appears to be triggered by seeing a happy expression on a female face [6]. In higher primates the auricular muscles are vestigial but seem to have a role in movements of the pinna. Some humans are able to voluntarily control the movements of their pinnae, and we aim to assess whether damage to this muscle group through postauricular incisions has an impact on this rudimentary function.

Some of the potential complications of postaural incisions have been investigated previously. Investigating cutaneous sensory deficit, Kang et al. [7] found that the sensation of the pinna returned to baseline within three months for sulcus incisions. However, a questionnaire-based study where the type of incision was not specified found that 26% of patients had persisting numbness beyond eight months [8]. Cosmetic outcomes have also been investigated. Retroauricular skin scars had an excellent aesthetic outcome in cochlear implant surgery [2]; and in a small follow up study, Hong et al. [9] noted that in 19 children a postauricular approach did not significantly affect pinna position. Shekhar and Bhavana [10] concluded that “behind the groove” incisions are better at preserving the conchomastoid angle than “in the groove” incisions and therefore give a better overall cosmetic result.

Little is known about the functional impact that a postauricular incision might have on the patient in daily life. Almost all patients will wear either sun glasses or vision correcting glasses at some stage during their postoperative lifetime, and the importance of a functional postauricular sulcus for eyeglass wearer has been highlighted [11, 12]. After ear surgery there is often an increased likelihood of needing to wear a hearing aid, and as behind the ear devices remain the most commonly used, there is a surprising lack of reports in the literature to examine patient comfort with this. In our study, we aimed to find out if patients perceived their “hairline scars” to affect comfort in wearing glasses and hearing aids.

## 2. Materials and Methods

### 2.1. Ethical Consideration

No ethics approval was required for this study. Approval was granted from the local Research and Development Department to carry out this questionnaire.

### 2.2. Study Design

The local Information services department provided the identities of all patients who underwent either “mastoidectomy,” “combined approach tympanoplasty,” or “cortical mastoidectomy” surgery between January 2004 and December 2012. These procedures all require drilling of the mastoid bone and therefore have the same incision site. Only procedures performed by or under direct supervision of one surgeon (DJW) were included. This information was derived from electronic theatre and admission records. No age restrictions were applied.

A printed paper questionnaire was designed to address the aims of the project, and the questions asked were as follows.

1. Do you experience any pain or difficulty wearing glasses?
2. Do you experience any pain or difficulty wearing a hearing aid?
3. As part of the operation a small group of muscles behind the ear are cut. The function of these muscles is not clear, but they may have a role in moving the external ear as seen in other animals. Were you able to move or “wiggle” your ears before the operation?
4. If so, has this movement been affected by the surgery?

Patients were asked to respond to each by highlighting “yes,” “no,” or “not applicable.” Additional space was provided after each question, and participants (including those who did not wear audiovisual aids regularly) were encouraged to provide their comments.

Contact details were acquired from the hospital database, and questionnaires were then posted to participants in January 2014 complete with a stamped and addressed envelope for return. The results were collated over a 4-week period after which no further questionnaires were returned.

## 3. Results

### 3.1. Participants

A total of 100 operations were identified in the initial search as meeting the inclusion criteria. The search criteria excluded “tympanoplasties” and “myringoplasties.” Whilst these can be performed through postaural incisions the authors favour an endaural incision for these procedures, and additionally the mastoid bone is usually preserved. Within the included operations, 5 patients had undergone bilateral surgery, 4 had a second look combined approach tympanoplasty, and further 4 patients had revision surgery or a conversion procedure (e.g., canal wall up converted to canal wall down). One patient was excluded at this stage as his operation was performed for acute mastoiditis and the incision site would therefore not be consistent with the other procedures.

A further 5 patients were excluded from questionnaires; 2 who had moved from the area but had not provided a forwarding address and 3 who were reported deceased on the hospital database.

In total, therefore, 81 questionnaires were posted. Completed responses were analysed for 42 patients (52%).

### 3.2. Wearing Glasses

In response to the first question relating to pain or difficulty wearing glasses 9 (21%) participants replied with “not applicable.” Of the 33 responders who wear glasses, 26 (79%) denied any pain or difficulty, whilst 7 (21%) reported that they did experience some degree of pain or difficulty. One patient commented as follows: “irritated of wearing sunglasses/3D glasses/safety glasses after prolonged use more so on scar,” whilst another replied as follows: “a little discomfort sometimes. Optician has to realign glasses on left due to indent in bone.” There were others who complimented the outcome including “no pain at all to do with the incisions.”

### 3.3. Wearing Hearing Aids

20 responders indicated that this question was not applicable. Of the 22 patients wearing hearing aids, 18 (82%) reported no pain or difficulty associated with their use. One responded as follows: “I am very certain that if I did wear an aid my scar would not be of any problem either. Super job done, thank you.” Four (18%) however did report problems including “still sore on the scar and finding it painful to keep hearing aid in longer than a couple of hours.”

### 3.4. Voluntary Pinna Movement

18 (43%) of participants did not know if they were able to voluntarily move their pinnae preoperatively. The majority (19, 45%) reported that they were unable to perform this function, whilst 5 (12%) reported that they were able to. Only one patient felt that the degree of movement was affected by the surgery, responding “I could “wiggle” both ears, but the ear operated on has lost a tiny bit of movement, approx. 15–20%.”

## 4. Discussion

*Key Results*.

- 79% of glasses wearers reported no pain or discomfort.
- 82% of hearing aid users reported no pain or discomfort.

*Limitations*. Inevitably with patient satisfaction questionnaires, several factors may bias the results. We tried to eliminate selection bias by contacting all patients who underwent surgery and fitted the time and operating consultant criteria. Response bias influences which patients respond to a questionnaire, and it is possible that we only had responses from patients who perceived their surgical episode to be either particularly good or particularly poor. Whilst a response rate of 50% is reasonable from a questionnaire we have to be cautious in speculating on the experiences of those who did not participate. We also acknowledge that racial demographics of the local population are quite different to some other areas of the country being almost exclusively Caucasian in this study, and therefore keloid scarring is unusual.

It would be useful to compare the patient satisfaction of this cohort with a group who have had a more anterior, sulcal incision. This would help establish which approach is more comfortable for patients.

Whilst all procedures were performed under the supervision of one consultant, the operating surgeon, particularly at the point of wound closure, will have varied from patient to patient. However in each case the same technique is used (interrupted deep sutures followed by continuous subcutaneous wound closure with 4-0 absorbable synthetic polyfilament).

Finally it is acknowledged that at the time of participation in the questionnaire many patients were not regular glasses or hearing aid users, but with time they may become so. It is not possible to comment on whether they will have problems with their scars in the future.

### 4.1. Wearing Glasses

The position of the pinna can be affected by incisions used to access the mastoid [13], but the effect of this and the scar site itself are rarely considered. Our results suggest that generally a far posterior incision is compatible with comfort in wearing glasses. One patient commented on the change of alignment required as a result of the “indent.” Irrespective of the surgical approach, a mastoidectomy will leave this deficit, and it is worth considering this when consenting patients particularly those who are regular glasses wearers.

### 4.2. Wearing Hearing Aids

After mastoid surgery it is reasonable to expect a higher incidence of hearing aid use than that of the general population. Although there is a range of aids available, the most commonly worn are behind the ear aids. Most of these rely on the natural support of the pinna and sit close to the retroauricular sulcus so may cause more irritation to a scar in this area.

### 4.3. Voluntary Pinna Movement

The movements of the ear in humans cannot be isolated to the postauricular muscles alone as there are contributions from temporalis and frontalis. Whilst in humans the postauricular reflex is vestigial, it is possible to measure the contractions in these muscles using EMG. The fact that only one of the patients who could voluntarily move his pinnae preoperatively noticed a change in function after surgery would also imply that this muscle group is of limited importance, but the response rate and low study numbers mean that no robust conclusions can be drawn from this. It is possible that some muscle contractions are feasible with healing postoperatively, but no active attempt is made to close the muscle layers as functional units. It is more likely in this isolated case that the fibrosis and scarring affected the degree of fixation of the pinna onto surrounding tissues.

## 5. Conclusions

This survey has provided evidence that most patients who wear glasses and hearing aids can do so without discomfort from the far posterior postauricular incision. It would be interesting to compare these results with patients who have incisions closer to the retroauricular groove. Whilst infrequent but serious complications of middle ears surgery such as facial nerve injury are usually discussed as part of the preoperative consent process, we feel that patients should also be informed of the far more frequent problems they may face with wearing audio and visual aids as a result of the operation.
